# Symptom relief, prognostic factors, and outcome in patients receiving urgent radiation therapy for superior vena cava syndrome: A single-center retrospective analysis of 21 years’ practice

**DOI:** 10.1007/s00066-022-01952-z

**Published:** 2022-05-12

**Authors:** Manuel Guhlich, Teresa Esther Maag, Leif Hendrik Dröge, Rami A. El Shafie, Andrea Hille, Sandra Donath, Markus Anton Schirmer, Olga Knaus, Friedemann Nauck, Tobias Raphael Overbeck, Marc Hinterthaner, Wolfgang Körber, Stefan Andreas, Achim Rittmeyer, Martin Leu, Stefan Rieken

**Affiliations:** 1 Clinic of Radiotherapy and Radiation Oncology, University Medical Center Göttingen, Göttingen, Germany; 2 Department of Palliative Medicine, University Medical Center Göttingen, Göttingen, Germany; 3 Department of Hematology and Medical Oncology, University Medical Center Göttingen, Göttingen, Germany; 4 Lung Cancer Center, Medical University Göttingen and Göttingen Comprehensive Cancer Center (G-CCC), Göttingen, Germany; 5 Department of Thoracic and Cardiovascular Surgery, University Medical Center Göttingen, Göttingen, Germany; 6 Pneumology Department, Evangelisches Krankenhaus Göttingen-Weende gGmbH, Göttingen, Germany; 7 Lungenfachklinik Immenhausen, Immenhausen, Germany

**Keywords:** Superior Vena Cava Syndrome, Radiotherapy, Symptom relief, Retrospective

## Abstract

**Purpose:**

Superior vena cava syndrome (SVCS) often results from external vessel compression due to tumor growth. Urgent symptom-guided radiotherapy (RT) remains a major treatment approach in histologically proven, rapidly progressive disease. Despite several publications, recent data concerning symptom relief and oncological outcome as well as potential confounders in treatment response are still scarce.

**Methods:**

We performed a retrospective single-center analysis of patients receiving urgent RT between 2000 and 2021 at the University Medical Center Göttingen. Symptom relief was evaluated by CTCAE score during the RT course. Effects of variables on symptom relief were assessed by logistic regression. The impact of parameters on overall survival (OS) was evaluated using Kaplan–Meier plot along with the log-rank test and by Cox regression analyses. Statistically significant (*p*-value < 0.05) confounders were tested in multivariable analyses.

**Results:**

A total of 79 patients were included. Symptom relief was achieved in 68.4%. Mean OS was 59 days, 7.6% (*n* = 6) of patients showed long-term survival (> 2 years). Applied RT dose > 39 Gy, clinical target volume (CTV) size < 387 ml, concomitant chemotherapy, and completion of the prescribed RT course were found to be statistically significant for OS; applied RT dose and completion of the prescribed RT course were found to be statistically significant for symptom relief.

**Conclusion:**

Symptom relief by urgent RT for SVCS was achieved in the majority of patients. RT dose and completion of the RT course were documented as predictors for OS and symptom relief, CTV < 387 ml and concomitant chemotherapy were predictive for OS.

**Supplementary Information:**

The online version of this article (10.1007/s00066-022-01952-z) contains supplementary material, which is available to authorized users.

## Introduction

Superior vena cava syndrome (SVCS) comprises several symptoms associated with obstruction of the superior vena cava. Obstruction is mainly caused by compression and/or invasion of the superior vena cava (SVC) due to malignant tumor growth [1]. Nonmalignant causes primarily include thrombosis of the SVC or benign tumor growth, e.g., benign thymoma [2].

In slowly developing cases, collateral pathways such as the azygos–hemiazygos pathway may prevent the patient from developing severe symptoms [3]. However, in rapidly evolving SVCS, e.g., due to massive tumor growth, most patients show distinctive symptoms [3, 4]. These include, but are not limited to, shortness of breath (caused by compression or laryngeal and/or tracheal edema) and swelling of neck and face (due to increased blood volume and lymphostasis) [5]. Prolonged and/or rapidly increasing untreated SVCS can lead to cerebral edema, causing headache, confusion, apathy, and can ultimately lead to death [2, 6, 7]. Different authors have developed scoring systems [8, 9] helping to determine the need for (urgent) treatment. In contemporary literature, emergency treatment is generally deemed a reluctant approach, especially if the underlying cause of SVCS is not yet determined [4, 10, 11]. However, clinical deterioration can progress quickly in severe cases. Therefore, SVCS has historically been [12, 13] and in many cases still is assessed as an oncological emergency situation [14, 15].

Histological examination is mandatory to prioritize treatment strategies. Symptomatic approaches include orthostatic positioning, administration of glucocorticoids, supplementation of oxygen, and, in severe cases of dyspnea, opioids [16]. Chemotherapy, endovascular stenting (ES), and immediate radiotherapy (RT) are common treatment options [4, 9, 17, 18]. Despite recent publications promoting the use of ES, this procedure might be either unavailable locally or infeasible, e.g., due to the presence of endovascular catheters [19]. Therefore, urgent RT remains a relevant treatment regime. Due to a lack of prospective studies [20], questions concerning its palliative effectiveness, RT dose, and RT technique schemes remain unanswered. Furthermore, and despite the sudden onset of urgent symptoms, a few patients with SVCS are in limited disease stages and may be candidates for curative therapies. Data concerning the treatment of these patients are currently missing. We, therefore, performed this retrospective analysis.

## Patients and methods

### Patients and study design

This single-center retrospective study includes patients treated at the Department of Radiotherapy and Radiooncology at the University Medical Center in Göttingen, Germany, between 2000 and 2021. Patients and their respective diagnoses were identified by systematic keyword screening for “vena cava syndrome.” Data and follow-up data were extracted from physical patient records and radiotherapy treatment planning systems (Varian Eclipse, version 15.6, Varian Medical Systems, Palo Alto, USA). Patient follow-up was evaluated through screening of hospital intern data processing systems (ixserv.4, version R20.3, ix.mid software technology, Köln, Germany) and ONKOSTAR (version 2.9.8, IT-Choice Software AG, Karlsruhe, Germany). The study was conducted according to the guidelines of the Declaration of Helsinki and approved by the Ethics Committee of the University of Göttingen Medical Center (protocol code 19/5/21, date of approval: 07 June 2021).

**Fig. 1:**
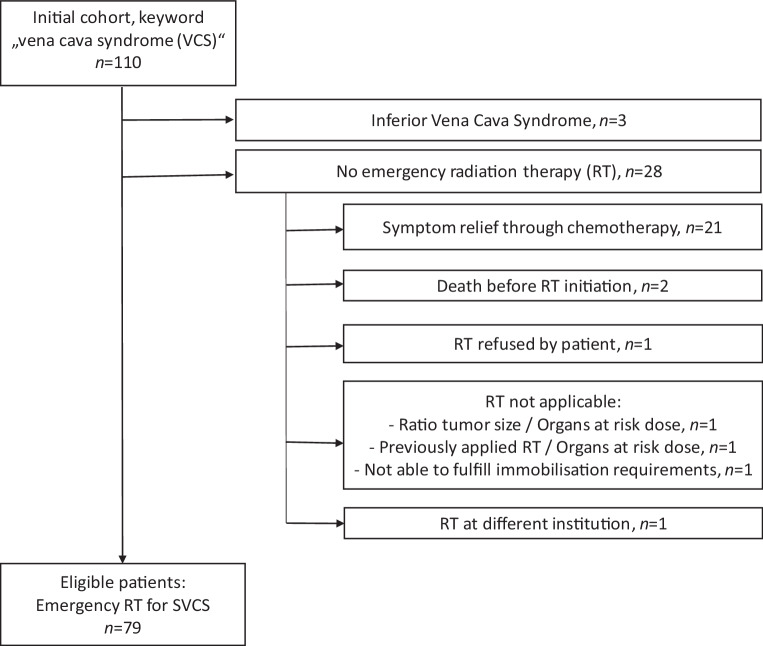
Flowchart of patient selection. Initial screening by keyword “vena cava syndrome” in patient-processing data systems in years 2000–2021

A total of 79 patients were eligible for analysis. Please refer to Fig. 1 for patient selection.

Patient age ranged from 29 to 81 years. All patients had histologically proven disease before RT start. Predominant diagnoses were small cell lung cancer (SCLC, *n* = 24, 30.4%) and non-small cell lung cancer (NSCLC, squamous cell carcinoma, *n* = 23, 29.1%; adenocarcinoma, *n* = 16, 20.2%; large cell neuroendocrine carcinoma, *n* = 3, 3.8%). A majority of patients had a strong history of smoking (*n* = 51, 64.6%), 11 of those (13.9%) combined with alcohol abuse. Charlson comorbidity index (CCI) was evaluated for all patients (range 1–3: 25.3%; 4–6: 40%; 7–10: 36.7%). Please see Table 1 for baseline patient and treatment characteristics. Table 2 comprises details on treatment and side effects, Table 3 on applied RT dose and fractionating scheme, EQD_2_ and BET_10_ equivalents for included patients, as well as treatment-related remarks.

**Table 1:** Baseline patient and disease characteristics

**Patient characteristics**	
*Patients, N (%)*	79
*Age (years), median (min–max)*	62 (29–81)
*Sex: female:male, N (%)*	30 (38) : 49 (62)
*Charlson comorbidity index, N (%)*	
1–3	20 (25.3)
4–6	30 (40.0)
7–10	29 (36.7)
**Disease characteristics**	
*SVCS as first symptom of disease, N (%)*	22 (27.8)
*Histology, N (%)*	
SCLC	24 (30.4)
NSCLC: SCC	23 (29.1)
NSCLC: adenocarcinoma	16 (20.2)
NSCLC: large cell neuroendocrine carcinoma	3 (3.8)
Other entities^a^	13 (16.5)

*SVCS* superior vena cava syndrome, *SCLC* small cellular lung cancer, *NSCLC* non-small cellular lung cancer, *SCC* squamous cell carcinoma

^a^Other entities: breast cancer (*n* = 3), non-Hodgkin lymphoma (*n* = 2), thymoma (*n* = 2), sarcoma (*n* = 2), Hodgkin’s lymphoma (*n* = 1), renal cell carcinoma (*n* = 1), unable to differentiate between adenocarcinoma and SCC (*n* = 1), mixed-cell: SCC and SCLC (*n* = 1)

**Table 2:** Treatment details and treatment related toxicity

**Radiotherapy (RT) technique**^**a**^**, *N* (%)**	
2D RT	1 (1.3)
3D conformal RT	64 (81.0)
IMRT	3 (3.8)
VMAT	11 (13.9)
Dose, median (min–max)^b^	39.0 Gy (3–66)
**Course of RT, *N* (%)**	
Intended RT complete	55 (69.6)
Intended RT incomplete	24 (30.4)
Death during RT	17 (21.5)
*Symptom relief:* all patients	54 (68.4)
*Symptom relief:* patients with intended RT complete	49 (89.1)
Change to curative concept	13 (16.5)
**Target volume (TV) features**	
Size of CTV (ml), median (min–max)^c^	387 (89.5–1966.2)
Size of PTV (ml), median (min–max)^c^	992.9 (288.5–3659.6)
TV adjusted during therapy*, N *(%)	18 (22.8)
Adjusted TV smaller than initial TV *N* (%)	10 (55.6 referring to above)
**Acute treatment-related side effects**^d^**, *N* (%)**	
Grade 1	30 (38.0)
Grade 2	7 (8.9)
Radiation induced pneumonitis	2 (2.5)
**Systemic therapy, *N* (%)**	
Concomitant chemotherapy	15 (19.0)
Chemotherapy, any	53 (67.1)
Immunotherapy, any	8 (10.1)

*IMRT* intensity-modulated radiotherapy, *VMAT* volumetric modulated arc therapy, *TV* target volume, *CTV* clinical target volume, *PTV* planning target volume, *RT *radiotherapy

^a^High proportion of 3D conformal RT due to fast planning approach in order to deliver rapid treatment (usually, 2‑3 h after consulting the patient)

^b^Radiotherapy for superior vena cava syndrome only. For details, refer to Table 3

^c^Not applicable in 1 patient due to 2D radiotherapy (see above)

^d^Acute toxicity as scored by Common Terminology Criteria for Adverse Events, v.5 [21]. There was no toxicity exceeding grade 2

**Table 3:** Details concerning applied RT dose and fractionating scheme for all patients of the study (*N* = 79) with corresponding EQD_2_ (α/β:10) and BED_10_

Applied dose (Gy)	1st fractionation (fractions * Gy)	2nd fractionation (fractions * Gy)	3rd fractionation (fractions * Gy)	EQD_2_ (α/β:10)	BED_10_	Patients, *N* (%)	Comment
3	1 * 3	–	–	3.25	3.9	1 (1.3)	RT aborted prematurely
6	2 * 3	–	–	6.5	7.8	2 (2.6)	RT aborted prematurely
6	3 * 2	–	–	6	7.2	1 (1.3)	RT aborted prematurely
9	3 * 3	–	–	9.75	11.7	1 (1.3)	RT aborted prematurely
11	3 * 3	1 * 2	–	11.75	14.1	1 (1.3)	RT aborted prematurely
12	4 * 3	–	–	13	15.6	1 (1.3)	RT aborted prematurely
15	5 * 3	–	–	16.25	19.5	1 (1.3)	RT aborted prematurely
17	3 * 3	4 * 2	–	17.75	21.3	1 (1.3)	RT aborted prematurely
19	3 * 3	5 * 2	–	19.75	23.7	1 (1.3)	RT aborted prematurely
24	8 * 3	–	–	26	31.2	1 (1.3)	RT aborted prematurely
20	10 * 2	–	–	20	24	1 (1.3)	Low dose due to Re-Irradiation
20.4	2 * 3	8 * 1.8	–	20.66	24.79	1 (1.3)	Low dose due to Re-Irradiation
25	3 * 3	8 * 2	–	25.75	30.9	1 (1.3)	RT aborted prematurely
27	5 * 3	6 * 2	–	28,25	33.9	1 (1.3)	RT aborted prematurely
27	9 * 3	–	–	29.25	35.1	1 (1.3)	RT aborted prematurely
29	3 * 3	10 * 2	–	29.75	35.7	1 (1.3)	RT aborted prematurely
30	10 * 3	–	–	32.5	39	3 (3.6)	–
31	3 * 3	11 * 2	–	31.75	38.1	2 (2.6)	RT aborted prematurely (*n* = 1)
33	3 * 3	12 * 2	–	33.75	40.5	1 (1.3)	RT aborted prematurely
36	20 * 1.8	–	–	35.4	42.48	1 (1.3)	–
36	12 * 3	–	–	39	46.8	1 (1.3)	RT aborted prematurely
39	13 * 3	–	–	42.25	50.7	14 (17.7)	–
39	5 * 3	12 * 2	–	40.25	48.3	1 (1.3)	–
39	3 * 3	15 * 2	–	39.75	47.7	1 (1.3)	–
39.6	22 * 1.8	–	–	38.94	46.73	1 (1.3)	Curative concept (*n* = 1, NHL)
40	20 * 2	–	–	40	48	1 (1.3)	–
41	3 * 3	16 * 2	–	45.75	54.9	1 (1.3)	RT aborted prematurely/curative concept intended (*n* = 1, NSCLC)
42	21 * 2	–	–	42	50.4	2 (2.6)	RT aborted prematurely
44	22 * 2	–	–	44	52.8	3 (3.6)	–
45	3 * 3	18 * 2	–	45.75	54.9	8 (10.1)	Curative concept (*n* = 2, SCLC)
45	15 * 3	–	–	48.75	58.5	1 (1.3)	–
45	25 * 1.8	–	–	44.25	53.1	1 (1.3)	Curative concept (*n* = 1, SCLC)
46	23 * 2	–	–	46	55.2	1 (1.3)	RT aborted prematurely/curative concept intended (*n* = 1, NSCLC)
49	3 * 3	20 * 2	–	49.75	59.7	2 (2.6)	–
50	25 * 2	–	–	50	60	5 (6.3)	Curative concept (*n* = 1, Thymoma)
53	3 * 3	22 * 2	–	53.75	64.5	1 (1.3)	–
59	3 * 3	25 * 2	–	59.75	71.7	5 (6.3)	Curative concept (*n* = 2, NSCLC)
59.4	3 * 3	28 * 1.8	–	59.31	71.17	1 (1.3)	Curative concept (*n* = 1, NSCLC)
60.4	3 * 3	5 * 2	23 * 1.8	60.46	72.55	1 (1.3)	–
65	3 * 3	28 * 2	–	65.75	78.9	1 (1.3)	Curative concept (*n* = 1, Thymoma)
66	33 * 2	–	–	66	79.2	3 (3.6)	Curative concept (*n* = 3, NSCLC)

*SCLC* small cell lung cancer, *NSCLC* non-small cell lung cancer, *NHL* non-Hodgkin lymphoma

### Endpoints

As SVCS is considered an indication for immediate symptom-directed RT, symptom relief in terms of subjective and/or objective reduction of primarily presented clinical expression of SVCS was chosen as the primary endpoint. The endpoint was defined as follows: a relevant reduction of subjective dyspnea or objective oxygen demand, decline or absence of initial cervical vein congestion in imaging analyses, clinical decline or absence of initial swelling of neck and face, clinical decline or absence of initial stridor. The primary endpoint of symptom reduction was considered achieved when all of the above-listed symptoms were reduced to a maximum of Common Terminology Criteria for Adverse Events (CTCAE) grade 1, if applicable (v.5.0, [21]). Morbidities corresponding to CTCAE > grade 1 were chosen as any morbidity above 1 shows distinct limitations to activities of daily living. Patients were monitored on a daily basis during emergency treatment, including a thorough clinical examination and imaging examinations for setup control during RT. Treatment effects, potential treatment-related side effects and laboratory results were documented at least once a week and reviewed by experienced radiation oncologists. Secondary outcomes were overall survival (OS), tumor-specific survival (TSS), and treatment-related toxicities, calculated from the beginning of RT until death or onset of toxicity.

### Statistical analyses

Data were analyzed using the software SPSS (v. 26; IBM Corp., Armonk, NY, USA) and R (v. 4.0.2; R: a language and environment for statistical computing. R Foundation for Statistical Computing, Vienna, Austria, https://www.R-project.org/) with the “KMWin” (Kaplan–Meier for Windows) plugin [22]. Survival statistics were evaluated using the Kaplan–Meier estimator. Survival times were compared using log-rank tests. Univariable cox regression was applied for assessing the impact of variables on survival, univariable logarithmic regression likewise with regard to symptom relief. We considered *p*-values < 0.05 as statistically significant. Univariably significant variables were also tested in a multivariable fashion.

## Results

### Symptom relief from SVCS after radiotherapy

In total, 79 patients were eligible for analysis. Symptom relief as described in section “Endpoints” by RT treatment was achieved in 54 patients (68.4%). No patient received an SVC stent placement before RT. Seventeen patients (21.5%) died during therapy, 7 patients (8.9%) did not finish RT (*n* = 3 due to significant deterioration of general condition, *n* = 3 due to patients’ choice, *n* = 1 due to newly diagnosed hepatic metastases). When considering only patients who completed the intended RT regime, 49 out of 55 patients (89.1%) showed a significant symptom relief.

To evaluate different prognostic factors for patients’ symptom relief, we performed a logistic regression. Applied RT dose and completion of intended RT course remained statistically significant in a multivariable model (Table 4).

**Table 4:** Influence of potential prognostic factors on patients’ symptom relief

Variable	Symptom relief		
	Hazard ratio (95% CI)	*P*-value univariable	*P*-value multivariable
Age	0.96 (0.96–1.03)	0.74	–
Sex	1.46 (0.53–3.97)	0.46	–
CCI	0.71 (0.54–0.92)	< 0.09*	n. s.
Applied dose in Gy	1.13 (1.07–1.20)	< 0.01*	0.01*
CTV in ml	1.00 (0.99–1.02)	0.75	–
Concomitant CTx	1.91 (0.53–8.22)	0.29	–
Prescribed RT complete	39.0 (7.69–197.70)	< 0.01*	0.02*

Calculations were done by logistic regression analyses. *P*-values < 0.05 were considered statistically significant. Variables with *p* < 0.1 in univariable analysis were consecutively tested in a multivariable logistic regression model

*CI* confidence interval, *CCI* Charlson comorbidity index, *CTV* clinical target volume, *CTx* chemotherapy, *n.* *s.* not significant

*Statistically significant *p*-value

Symptom-wise, a subgroup of patients (*n* = 6) did not profit from palliative RT despite completing the prescribed RT. When analyzing these patients in terms of disease characteristics, lifestyle factors, RT course, RT technique, and treatment-related side effects as well as treatment compliance, no significant prognosticator was identified. Notably, as this turned out statistically influential on patients’ overall survival (see Table 5), all of the patients had a CTV size < median.

### Overall survival

**Fig. 2:**
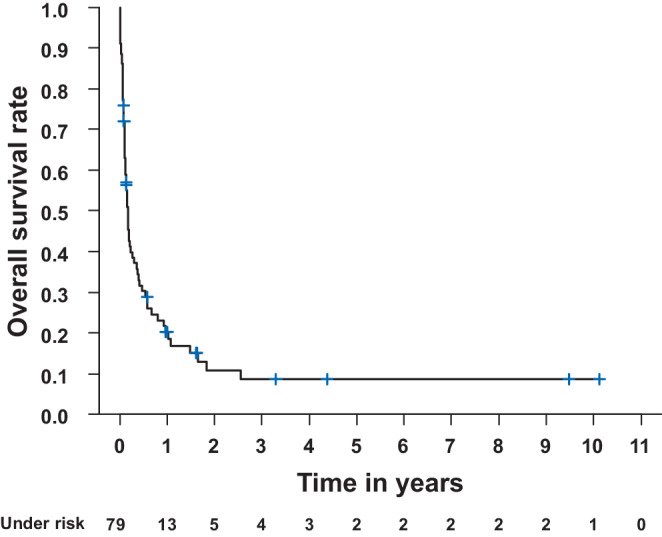
Kaplan–Meier estimate of all patients

Patients’ median overall survival (OS) was 59 days (range 2–3691; Fig. 2). Additional Kaplan–Meier estimates stratified by histology are provided in the supplementary material (n. s.). We evaluated the following different variables to determine a potential influence on patients’ OS: age, sex, Charleston comorbidity index (CCI), applied RT dose, size of clinical target volume (CTV), concomitant chemotherapy, and completion of intended RT course. Statistically significant *p*-values (each ≤ 0.01) were found for CCI (worse OS for more comorbidities, hazard ratio [HR] 2.05), delivered RT dose (better OS for patients receiving > 39 Gy, HR 0.32), size of CTV (worse OS for larger CTVs, HR 1.91), concomitant chemotherapy (better OS, if concomitant chemotherapy administered, HR 0.38), and completing the intended RT course (better OS if prescribed dose was reached, HR 0.27). For details, please refer to Table 5.

**Table 5:** Influence of potential prognostic factors on patients’ OS

Variable (*n*)	Overall survival		
	Hazard ratio (95% CI)	*P*-value univariable	*P*-value multivariable
*Age* *per year*	1.01 (0.99–1.03)	0.28	–
≥ 70 (20) vs. < 70 (59)	0.87 (0.51–1.49)	0.62	–
*Sex*			
Female (30) vs male (49)	0.76 (0.46–1.27)	0.30	–
*CCI*			
> 6 (29) vs. ≤ 6 (50)	2.05 (1.23–3.41)	< 0.01*	n. s.
*Dose in Gy*			
> 39 (38) vs. ≤ 39 (41)	0.32 (0.19–0.53)	< 0.01*	0.01*
*CTV in ml*			
> 387(38) vs. ≤ 387 (41)	1.91 (1.16–3.14)	0.01*	0.022*
*Concomitant CTx*			
Yes (15) vs. no (64)	0.38 (0.19–0.36)	< 0.01*	0.024*
*Prescribed RT complete*			
Yes (62) vs. no (17)	0.27 (0.01–0.07)	< 0.01*	< 0.01*

CCI and CTV were dichotomized by median. Calculations were done by univariable cox regression. *P*-values < 0.05 were considered statistically significant. Those variables with statistically significant *p*-values (*) in univariable analysis were consecutively tested in multivariable Cox regression

*CI* confidence interval, *CCI* Charlson comorbidity index, *CTV* clinical target volume, *CTx* chemotherapy, *n.* *s.* not significant

### Long-term survival

**Fig. 3:**
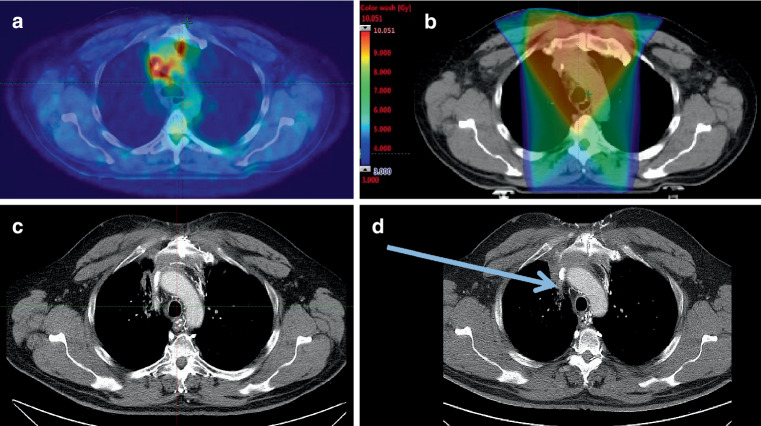
Example patient with long-term survival with initial emergency radiotherapy for superior vena cava syndrome and switch to curatively intended RT, histologically proven Masaoka stage III thymoma. **a** Axial slice of diagnostic positron-emission tomography (PET) scan, blended with RT planning CT ahead of treatment; **b** corresponding intensity-modulated radiotherapy planning for the first three fractions, dose color wash ranging from 1.0 Gy (*blue*, lowest value) to dose maximum (10,051 Gy, red) on this plane; **c** axial slice of diagnostic CT scan showing treatment response 6 months after RT; **d** axial slice of diagnostic CT scan showing ongoing remission 18 months after RT. Note the high PET avidity in the area of SVC on **a**, SVC detectable in **a**–**d** due to port catheter (*arrow* in **d**)

Notably, even though OS declines rapidly within the first year after RT, a small subgroup of patients (*n* = 6, 7.6%) experiences long-term survival, here defined as more than 2 years from the start of RT treatment. All analyzed patients started treatment with immediate RT for SVCS; 13 patients (16.5%, Table 2) were adjusted to a curative RT dose tailored to their primary diagnosis. This switch from palliative to curative treatment was evaluated when symptom relief was achieved, and adequate staging excluded distant metastasis. Of the 6 long-term survivors, *n* = 2 patients were diagnosed with Masaoka III thymoma (RT dose: 50, 65 Gy), *n* = 1 patient with UICC stage III SCC-NSCLC (RT dose: 66 Gy, concomitant cisplatin administered), *n* = 1 patient with UICC stage III adeno-NSCLC (RT dose: 66 Gy, concomitant cisplatin administered), *n* = 1 patient with SCC of unknown primary (SC-CUP, RT dose: 59 Gy, concomitant cisplatin administered), *n* = 1 patient with diffuse large B‑cell lymphoma (DLBCL, RT dose: 39.6 Gy). Four patients (66.7%) were still alive at last follow-up (*n* = 2 NSCLC, *n* = 2 thymoma), 1 (DLBCL) was lost to follow-up 29 months after RT start, 1 patient (SCC-CUP) died 30.5 months after RT initiation due to systemic progression. Fig. 3 gives an example of a long-term survivor who started emergency RT due to severe SVCS by thymoma changed to a curatively intended RT dose, and who was still in remission at last follow-up.

## Discussion

### Symptom relief

Overall, 89.1% of patients completing the prescribed RT dose experienced significant symptom relief to a maximum remaining CTCAE score of 1 (“asymptomatic or mild symptoms” [21]). When taking all patients who started the urgent radiation treatment into account, a total of 68.4% showed significant symptomatic relief. Due to the nature of SVCS, only retrospective data are available in the literature [20], mainly comprising small numbers of patients. In China 48 cases of varying malignant SVCS were reported to achieve symptom relief in only 50% (5/10 patients) in a radiotherapy alone and 54.5% (12/22 patients) in a chemoradiotherapy group [5]. RT doses ranged from 45 Gy/3-Gy fractions (fx) to 50 and above/2-Gy fx. Armstrong and colleagues reported on a large cohort of 125 patients with different primary tumors receiving RT with or without chemotherapy with good or excellent symptom relief in approximately 80%. Faster results were observed for patients starting with high single radiation doses (3–4 Gy/day for the first three fx vs. 2 Gy/d) [15]. This treatment regime was used in the majority of our cohort (Table 3). Lonardi et al. set up a study for the elderly (aged 70 and above) evaluating hypofractionated RT (2–3 × 6 Gy) for malignancy-associated SVCS, achieving symptom relief in 87% (*n* = 23) [23]. Another retrospective study showed up to 56% complete response (CR) and 96% partial response (PR) due to hypofractionated RT (*n* = 46) [24]. An analysis of 34 lung cancer patients receiving palliative RT reports an overall response rate of 85%, scoring 94% of patients with SCLC and 76% of patients with NSCLC [25]. A study group evaluating only limited-disease SCLC (LD-SCLC) presenting SVCS at initial diagnosis showed symptom relief in 87.7% (50/57 patients) by concurrent chemoradiotherapy [26]. Table 6 summarizes the current literature concerning radiotherapy for SVCS.

**Table 6:** Review of the literature concerning urgent RT for SVCS

Study: first author, year published	Journal	Patients included, *n*	Recruitment time	Median follow-up, months	Concomitant CTx included	Predominant tumor	Dose applied	Overall response rate
Armstrong, 1985 [15]	*Radiat. Oncol. Biol. Phys*	125	01/1965–12/1984	n. d.	Yes	79% BC	10–60 (Gy)	80%
Lonardi, 2001 [23]	*Support Care Cancer*	23	01/2000–03/2001	n. d.	No	65% BC	12–40 (Gy)	87%
Beck, 1990 [27]	*Strahlenther Onkol*	90	06/1983–11/1988	3.9	No	85% BC	20–60 (Gy)	27% PR or CR^a^
Rodrigues, 1993 [24]	*J. Lung Cancer*	46	1986–1992	8	No	82% BC	16–24 (Gy)	84% PR or CR^a^
Engelmeers, 1996 [25]	*Bull Cancer Radiother*	34	1986–1993	n. d.	No	100% BC	30–54 (Gy)	85%
Wang, 2015 [26]	*Int. J. Clin. Exp. Med*	57	01/2004–12/2009	19.6	Yes	100% SCLC	55.5–88 (BED)	88%
Davenport, 1976 [12]	*Cancer*	19	01/1971–07/1975	n. d.	No	84% BC	25–53 (Gy)^b^	89%
Mose, 2006 [28]	*Anticancer Res*	35	01/1997–12/2003	n. d.	Yes	91% BC	22–56.4 (Gy)	86%
Present study	*Strahlenther Onkol*	79	01/2000–12/2021	2	Yes	80% BC	3–66 (Gy)	68%

*CTx* chemotherapy, *n.* *d.* no data, *BC* bronchial carcinoma (both NSCLC and SCLC), *SCLC* small cell lung cancer, *BED* biologically effective dose, *PR* partial response, *CR* complete response, *RT* radiotherapy, *SVCS *superior vena cava syndrome

^a^No data concerning symptom relief, only state of remission documented

^b^Originally published in rads

By analyzing prognostic factors for patients’ symptom relief, we were able to demonstrate a statistically significant influence of the applied RT dose and completion of the prescribed RT course in a multivariable logistic regression model.

### Overall survival and long-term survival

Our patient cohort showed a mean overall survival of 59 days. This takes 17 patients into account who died during therapy and 7 further patients not completing the intended RT course.

None of the analyzed histologies showed a statistically significant impact on OS (Supplemental Fig. 1). A recent study evaluating outcome prediction in extensive-stage SCLC (ES-SCLC) presenting SVCS showed an OS for patients undergoing chemoradiotherapy of 13.3 months [29]. The authors of a retrospective analysis of 90 patients treated between 1983 and 1988 indicated for their cohort a prognostic effect of Karnofsky performance scale (KPS), radiation dose, and disease stage, showing no influence of previous treatments, age, or tumor grading [27]. Patients whose KPS was scored 50 or below had a median OS of only 17 days. Even though we did not evaluate KPS as it was not documented adequately, a retrospective KPS scoring of patients in our study dying early indicated they most likely scored ≤ 50%. The historically largest cohort of Armstrong et al. comprising 125 patients reported a median OS of 5.5 months [15]. Differences in OS might be due to a higher proportion of lymphoma patients in the aforementioned study (14% vs. 3.7% in our study), a disease known to respond very well to RT (5-year OS of 41% reported in [15]).

In our cohort, we report on 17 patients (21.5%) dying during RT and 7 (8.9%) aborting the intended RT course. These numbers appear high, and patients may have a better outcome if SVC stenting is implemented before the start of RT. Nevertheless, in a recent prospective phase II/III trial evaluating symptom relief after SVC stenting, 5/28 (19.7%) and 8/32 patients (25%) died within 30 days after stent implantation despite achieving a high symptom control rate, thus reflecting the overall poor prognosis of SVCS [18].

Irrespective of locally advanced, mostly malignant tumors being the cause of SVCS in our study cohort, 6 patients (7.6%) were able to achieve long-term survival of at least 2 years after RT initiation, 3 of whom received concomitant chemoradiotherapy. This survival is specifically notable as severe SVCS leading to urgent RT is often considered as having a dismal diagnosis. Armstrong et al. reported a 5-year OS of 41% in lymphoma and 5% for SCLC comprising SVCS as well as a 2-year OS of 2% for NSCLC in 1987 [15]. Retrospective data of 104 patients receiving treatment for malignant SVCS (about 54.4% receiving urgent RT) aimed at analyzing factors associated with OS suggested advanced age (> 50 years), history of smoking, and use of steroids to be associated with a poor outcome in univariable analysis. Certain primary malignancies, e.g., lymphoma, showed better OS. These factors, however, did not reach statistical significance in multivariable analysis [30]. In univariable analysis, our data suggest an influence of CCI, size of the CTV, concomitant chemotherapy, applied RT dose, and completion of the intended RT course. In multivariable testing, size of the CTV, concomitant chemotherapy, applied RT dose, and completion of the intended RT course remained significant OS influencers, which appears consistent with clinical experiences.

## Limitations and conclusions

The present study reports symptom relief and oncological outcomes with impacting variables identified in 79 patients receiving urgent RT for SVCS between 2000 and 2021 in a single center. Several limitations have to be considered regarding the reported data: Foremost, due to the analyzed subject, this study is of retrospective nature; therefore, uncontrolled factors may bias our results. Second, it comprises data of a single, albeit academic, center. Third, the number of patients enrolled was rather small, resulting in even smaller subgroups of different entities. Forth, patients receiving SVC stents were not included in this analysis.

Keeping these limitations in mind, we provide information concerning symptom relief, oncological outcome, and impacting factors in the treatment of SVCS by urgent RT. A wide majority of patients showed a quick and significant symptom relief (68.4%). In patients completing the intended RT course, 89.1% achieved symptom relief.

A statistically significant influence of the applied RT dose and completion of the RT course as prognostic factors for the most important palliative therapy aim, symptom relief, could be demonstrated. Furthermore, we report an effect on OS of the size of the CTV, applied RT dose, concomitant chemotherapy, and completion of the intended RT course. We describe a small subgroup (*n* = 6, 7.6%) of patients alive > 2 years after RT start, suggesting that long-term survival can be achieved, i.e., by adjusting the RT dose regimen from palliative to curative therapy regimes.

Compared to similar publications, this is one of the largest reported cohorts. For clinical implementation, we suggest RT doses of > 39 Gy, where applicable. Additionally, we provide helpful data for treatment- and outcome-related discussions with patients as well as with colleagues in multidisciplinary oncological and palliative care teams.

## Supplementary Information

Supplemental Fig. 1: Stratification of the influence of different primary entities on OS, not significant.
