# Supplementary material for: Symptom relief, prognostic factors, and outcome in patients receiving urgent radiation therapy for superior vena cava syndrome: A single-center retrospective analysis of 21 years’ practice
Source: Strahlenther Onkol. 2022 May 12;198(12):1072–81. doi: 10.1007/s00066-022-01952-z (PMC9700593; doi:10.1007/s00066-022-01952-z)
Supplement: Supplementary file 1 — Supplemental Fig. 1: Stratification of the influence of different primary entities on OS, not significant. [file 66_2022_1952_MOESM1_ESM.pdf]

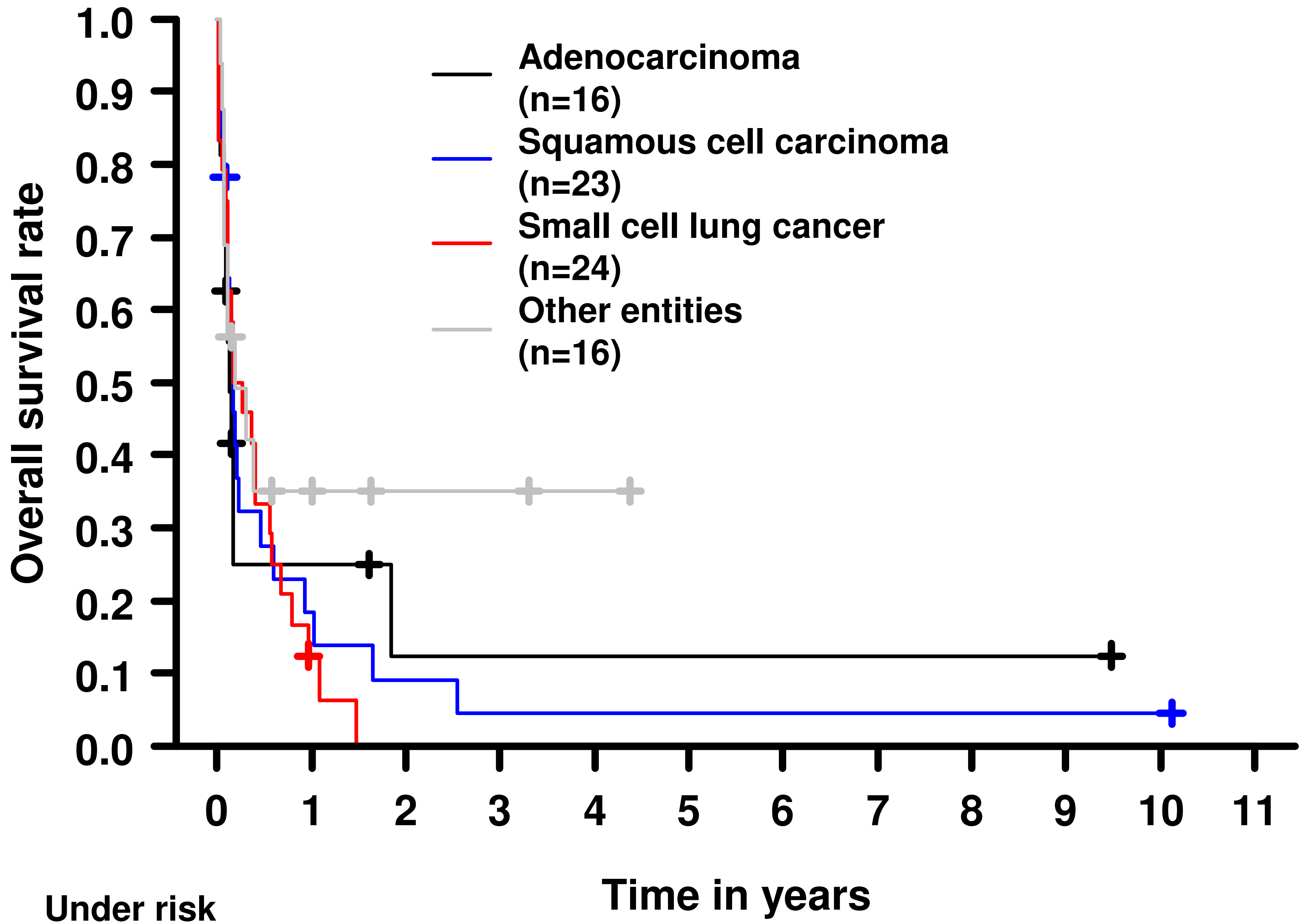

Under risk										
Adenocarcinoma	3	1	1	1	1	1	1	1	1	0	0
Squamous cell carcinoma	4	2	1	1	1	1	1	1	1	1	0
Small cell lung cancer	2	0	0	0	0	0	0	0	0	0	0
Other entities	4	2	2	1	0	0	0	0	0	0	0
